# Engineering Chimeric Antigen Receptors

**Published:** 2017

**Authors:** S. V. Kulemzin, V. V. Kuznetsova, M. Mamonkin, A. V. Taranin, A. A. Gorchakov

**Affiliations:** Institute of Molecular and Cellular Biology, SB RAS, Lavrentiev Ave. 8/2, Novosibirsk, 630090, Russia; Novosibirsk State University, Pirogova str. 2, Novosibirsk, 630090, Russia; Center for Cell and Gene Therapy, Baylor College of Medicine, Texas Children’s Hospital and Houston Methodist Hospital, Houston, TX, USA

**Keywords:** adoptive immunotherapy, cancer, chimeric antigen receptor, T cells

## Abstract

Chimeric antigen receptors (CARs) are recombinant protein molecules that redirect cytotoxic lymphocytes toward malignant and other target cells. The high feasibility of manufacturing CAR-modified lymphocytes for the therapy of cancer has spurred the development and optimization of new CAR T cells directed against a broad range of target antigens. In this review, we describe the main structural and functional elements constituting a CAR, discuss the roles of these elements in modulating the anti-tumor activity of CAR T cells, and highlight alternative approaches to CAR engineering.

## INTRODUCTION

Modern methods for re-targeting immune cells open unprecedented opportunities for the treatment of cancer and autoimmune diseases. Chimeric Antigen Receptors (CARs) represent one of the recent advances in this field. CARs are recombinant molecules that mediate cell activation upon encounter with the target antigen. The antigen-recognition domain of a CAR is typically derived from the sequences of monoclonal antibodies (mAbs). This domain functions to interact with tumor epitopes in an MHC-unrestricted manner. Cell activation is ensured by the signaling motifs in the intracellular portion of a CAR. At the moment, T cells are the most frequently used CAR “drivers” (CAR T cells), and this review focuses on the structural features of CARs, specifically in the context of T cells, although alternative cellular platforms exist, including NK cells, iNKT cells, and γδ T cells.

**Fig. 1:**
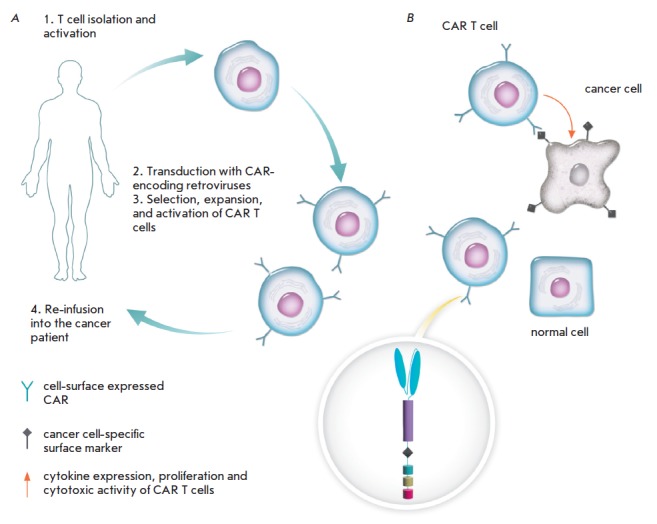
Adoptive cell transfer therapy with CAR T cells. Peripheral blood leukocytes are collected from a cancer patient in a process called leukapheresis. These cells are then stimulated ex vivo prior to transduction with CAR-encoding lentior retroviruses. Following this step, the transduced cells are selected, expanded, activated, and reinfused back into the patient (A). Upon encountering target cancer cells, CAR T cells become activated: they secrete cytokines, proliferate, and destroy cancer cells (B).

The outline of CAR T-cell therapy is shown in *Fig. 1*. First, a CAR-encoding DNA cassette is delivered into primary T cells collected from a patient. Next, transgenic CAR T cells are expanded *ex vivo *and re-infused into the patient, where they encounter target tumor cells. Tumor recognition is mediated by the antigen-recognition domain of a CAR, while its intracellular part induces T cell activation, which results in the destruction of tumor cells and proliferation of CAR T cells. Hence, this approach combines the selectivity of antibodies and the cytotoxic potential of T cells.

Although CAR T-cell therapy has only relatively recently transitioned from research laboratories into clinical trials, it has already shown highly promising results. Complete or partial remissions have been achieved in > 50% of leukemia patients that proved resistant to all other lines of therapy [1]. Meanwhile, the issues associated with the insufficient selectivity of CARs have also become apparent [2].

## CAR STRUCTURE

The CARs engineered in the mid-1980s encompassed variable fragments of antibodies fused with the constant regions of TCR α and β chains [3]. In 1993, Z. Eshhar and colleagues refined this design by using scFvs as antigen-recognition domains, whereas the transmembrane and signaling sequences were derived from CD3ζ or FcRγ; importantly, the entire chimeric receptor consisted of a single polypeptide chain [4]. Subsequent generations of CARs had an overall similar structure but also carried additional signaling modules for enhancing T-cell activity. The key structural components of CARs are discussed below.

## THE ANTIGEN-RECOGNITION DOMAIN OF CAR

**The scFv format**

**Fig. 2:**
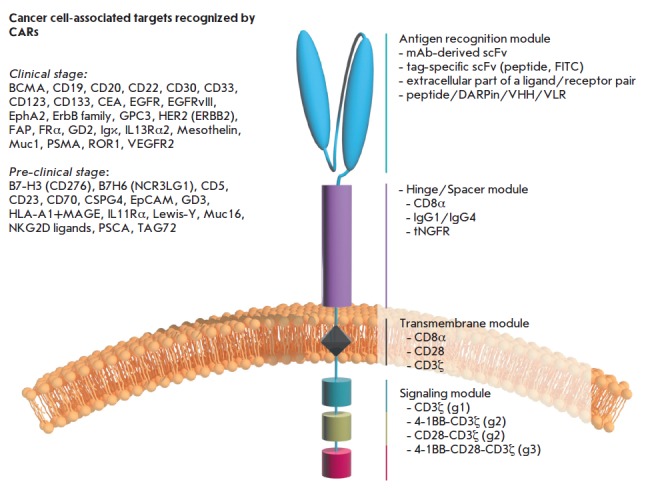
СAR structure (monomeric layout). First- (g1), second- (g2), and third- (g3) generation CARs differ in the number of costimulatory domains.

The vast majority of CARs use scFvs as antigen-binding modules [5] (*Fig. 2*). It is a convenient format, since the mAbs used for scFv design are typically well-characterized in preclinical models or have been approved for clinical use. Hence, the risk of unexpected cross-reaction between CAR T cells and healthy tissues is much lower although not absent when using CARs based on the previously tested scFvs. In addition, structural data are also frequently available for these antibodies, making it possible to change the affinity of scFv-based CARs in either direction in a targeted manner. However, the drawbacks of using scFvs as antigen-recognition domains in CARs include the risk of developing an immune response against the murine and linker sequences within scFv [6] and the difficulties in designing polyspecific scFv-based CARs because of their large size and the requirement for structure stabilization via disulfide bonds [7]. Furthermore, the framework sequences of antibodies within scFvs have been reported to induce ligand-independent CAR clustering, which results in tonic signaling, nonspecific activation, and, ultimately, premature exhaustion and loss of activity by CAR T cells. A. Long and colleagues tested several scFv-based CARs (targeting CD19, GD2, CD22, and HER2) for ligand-independent signaling and showed that only the CD19-specific CAR completely lacked this unwanted feature [8].

**Natural ligand–receptor pairs**

Most clinically tested CARs encompass non-humanized murine scFv sequences. This is associated with the risk of an immune response against CAR T cells and anaphylactic reactions [9] and may, thereby, compromise the efficacy of the CAR therapy. It is partially for this reason that alternative designs of the antigen-recognition moieties of CARs are being actively explored based on natural human ligand-receptor pairs. For example, the expression of an IL13 receptor, IL13Rα2, is often increased on the surface of glioblastoma, ovarian, and pancreatic cancer cells [10, 11]. Using this information, IL13-based CARs exhibiting specific recognition of IL13Rα2 were designed, although they were later found to recognize IL13α1 as well. [12-15]. Antigen-recognition domains of CARs specific to NKG2D ligands and CD70 have been designed using the extracellular domains NKG2D and CD27, respectively [16-18]. CARs recognizing HER3 (ErbB3) and HER4 (ErbB4) have been successfully produced by grafting the extracellular sequences from neuregulin 1 α and 1β [19, 20]. Finally, CARs containing sequences from CD4 [21-23], VEGF [24], and NKp30 [25], as antigen-recognition domains (specific for HIV gp120, VEGFR2, and B7H6, respectively), have been engineered.

It should be mentioned that, in general, CARs based on the ligand–receptor interplay have the same shortcoming as scFv-based CARs: the targets of these receptors are not entirely tumor-specific and are present, although at lower levels, on the surface of normal cells. Moreover, it is becoming progressively clear that receptors and ligands rarely have a single partner: usually there are several. Therefore, to eliminate the possibility of an unintended activation of CAR T cells after their encounter with cells expressing such off-target molecules, significant optimization of the CAR structure and function may be needed.

**Peptide ligands**

Peptide ligands have been successfully used as antigen-recognition domains in CARs. Despite their potential immunogenicity, peptides have an overall lower risk of triggering an immune response than much larger scFvs. D.M. Davies and colleagues designed the CAR containing peptide ligand T1E as an extracellular domain which recognizes target cells with surface expression of ErbB receptors [26]. Pameijer and colleagues showed that the 12-meric BPEP peptide within CAR enables successful recognition and destruction of target ovarian cancer cells expressing αvβ6 integrin [27]. A similar design has successfully been tested for the pair IL11Rα/nonapeptide IL11 (IL11Rα is typically overexpressed on osteosarcoma, gastric, intestinal, breast, and prostate cancer cells) [28]. At present, such peptide-based CARs are still in the proof of concept stage or undergoing preclinical validation.

A related approach is to use CARs whose antigen-recognition domain consists of designed ankryrin repeat proteins (DARPins) [29, 30], nanoantibodies (VHH) [31-34], or variable lymphocyte receptors (VLRs) [35]. DARPins are compact and stable protein modules selected for high-affinity binding to one or several targets. For instance, it has been shown that HER2-specific DARPin-CARs function (i.e., induce activation and cytotoxic reaction) comparably to “conventional” scFv-CARs against the same target. The functionality of VHHs and VLRs as antigen-recognition domains in CARs has also been described. The key advantages of this system include the modularity and smaller size of DARPins/VHHs/VLRs compared to that of scFvs, which in turn opens an exciting opportunity to design polyspecific and/or polyvalent CARs that can simultaneously recognize several targets. Nevertheless, the declared low immunogenicity of DARPin/VHH/VLR-CARs still remains to be demonstrated. This may lead to complications in the translation of such platforms into a clinical setting.

**Universal antigen-recognition modules**

Tumor cells are known to be typically quite heterogeneous with respect to surface markers, and so CAR T cells can recognize them with different efficiencies: CAR T cells will likely ignore cells that have downregulated or silenced the expression of the target surface molecule. Hence, it is tempting to design CAR T cells whose activity can be relatively easily re-targeted using an extensive toolbox of the available mAbs. Three design variants of the so-called universal antigen-recognition modules of CARs have been reported thus far.

The first variant uses the dimeric form of chicken avidin, the protein known to have high-affinity binding to biotin and biotinylated molecules, as an antigen-recognition domain of a CAR [36]. Infusion of these universal CAR (uCAR) T cells, along with biotinylated mAbs recognizing target tumor cells, results in efficient and specific eradication of tumor cells in mice. Furthermore, sequential infusion of biotinylated mAbs against other targets results in appropriate retargeting of uCAR T cells. Interestingly, free biotin, which is invariably present in blood plasma, does not appear to compromise this effect, nor does it cause nonspecific autoactivation of uCAR T cells. Likewise, the use of scFv-based CARs against a neoepitope peptide in combination with target-specific antibodies containing this neoepitope [37] allows one to obtain functional uCAR T cells.

The second variant of uCARs was based on the use of FITC-specific scFvs. The principle by which anti- FITC-CAR T cells function is similar to that described above: these cells recognize FITC-conjugated mAbs or scFvs, and therefore start recognizing and destroying the cells tagged with these molecules [38, 39].

Finally, the effect of CAR T cells mimicking NK cells, which can exhibit potent ADCC against malignant or infected cells, was used in the third system of universal CARs. As soon as an antibody binds to the surface of the target cell, its Fc region is recognized by CD16a (FcγRIIIA). Approximately 40% of people are known to carry the F158V polymorphism in CD16a, which significantly increases the affinity of this receptor to antibodies [41, 42]. The use of the extracellular domain of this receptor as the antigen-recognition domain made it possible to design uCARs that can re-target the cytotoxic activity of T cells according to the antitumor antibodies being infused [43-45]. This approach is potentially complicated by the presence of an excess of free antibodies present in the serum that may outcompete the administered mAbs in binding to CD16-CAR T cells. The results of clinical trials of CD16- CAR T cells, in combination with rituximab (anti-CD20 mAbs) in patients with CD20-positive non-Hodgkin’s lymphomas and chronic lymphocytic leukemia, will demonstrate whether this is, indeed, the case.

Hence, the above-described “universal” solution has two key benefits: (i) it is convenient to control uCAR CAR T cell specificity (i.e., to change it if necessary or to simultaneously infuse several antibodies against different targets) and (ii) these cells can be easily “switched off” by simply discontinuing the infusion of antibodies.

On the other hand, the potential immunogenicity of avidin, neoepitope peptides, and FITC may impede the smooth clinical translation of these CARs. This may be not too much of an issue, as many cancer patients are typically heavily immunosuppressed. Another problem is associated with the limited penetration of mAbs into organs and tissues, which may considerably reduce their effective concentration in solid tumors. In other words, these systems seem to suffer from the same drawbacks in the context of solid tumors as mAb-based therapies.

**The hinge module of CARs**

When a T cell interacts with an antigen-presenting cell, an immunological synapse with an intermembrane distance of ~15 nm is formed [46]. This distance is dictated by the architecture of TCR and the peptide–MHC complex. It determines the closed structure of the synapse and ensures physical exclusion of molecules that have extracellular domains longer than 15 nm. It turned out that this spatial separation is important for effective triggering of the phosphorylation cascade and T-cell activation [47, 48]. Thus, CD45 phosphatase has a bulky extracellular domain. When artificially shortened, this protein gets a chance to stay within the synapse, resulting in the suppression of activation signals [49, 50]. The distance between a CAR T cell and a tumor cell may be crucial in ensuring adequate activation of effector functions. Since mutual arrangement of the epitope on the target molecule and the antigen-recognition domain of the CAR in the context of the CAR T cell specifies the size of the synapse being formed, it becomes clear why this design feature can determine whether or not the CAR will be functional [51, 52]. For example, A.A. Hombach and colleagues demonstrated that CAR T cells recognizing the membrane-distal epitope of carcinoembryonic antigen (CEA) were moderately activated, while the same antigen transferred into a more proximal position resulted in much stronger CAR T cell activation [53]. Similarly, CAR T cells with the scFv recognizing a membrane-proximal epitope of CD22 (the antigen abundantly present on normal and malignant B cells) had high antileukemic activity, as opposed to the CAR T cells targeted against the membrane-distal epitope [54, 55]. These and some other examples [56, 57] indicate that membrane-distal epitopes in general tend to form synapses larger than the optimal 15 nm, and so this becomes compatible with the inclusion of CD45 and CD148 phosphatases, which in turn may attenuate the activation signaling.

Hence, given that the position of the epitope recognized by a specific scFv on the target cell surface is always fixed, the length and rigidity of the extracellular spacer (the hinge module) in the CAR needs to be adjusted empirically to ensure maximum steric compatibility with the scFv and the formation of a compact synapse.

CD8a, CD28, and IgG1/IgG4 (hinge-Fc part) sequences (in single studies, CD4, CD7, and IgD) are used most commonly as a spacer [58-61], review [62]. This choice is based on the fact that these sequences are relatively neutral, flexible, and have been well-characterized structurally. Nevertheless, the CD8a hinge has been reported to perform poorly in the context of certain scFv-based CARs, whereas the Fc-fragment of IgGs is far from biologically inert, and this has become apparent in *in vivo *studies. It was demonstrated that mutual recognition of cells with IgG-containing chimeric receptors and cells expressing Fc receptors (macrophages, monocytes, and NK cells) takes place. Specifically, IgG-CAR T cells become nonspecifically activated in the absence of the target antigen and attack FcRγ+ cells, which in their turn are activated and destroy IgG-CAR T cells, thereby influencing therapy efficacy and safety [63, 64]. One of the ways to address this problem is to use mutant IgG hinge variants that do not bind Fc receptors (with either a CH_2_-domain deletion or mutations in the key amino acid residues responsible for FcR binding) [63-66].

Interestingly, all the spacer variants being used in CARs are sequences prone to homo- or heterodimerizatiton; so, it is presently unclear whether the tonic/ ligand-independent signaling from these receptors helps or hinders CAR T cells. By default, dimerization is believed to contribute to the better surface retention of CARs [67]. *In vitro *data available demonstrate that CAR dimerization has little effect on the activation of CAR T cells [14, 68, 69], whereas *in vivo *experiments are needed to accurately compare the functionality of dimerizing and monomeric CARs. It must be noted that a CAR encompassing a spacer region derived from NGFR/p75 has been reported [70]: such a CAR will likely be ignored by nontarget cells and remain monomeric. Furthermore, the NGFR spacer can function as a convenient epitope, which may simplify the selection and expansion of CAR T cells, as well as help promptly destroy these CAR T cells in the patient’s body, once needed.

**Transmembrane module**

The transmembrane module functions to anchor the receptor on the cell surface. This domain usually includes the transmembrane sequences of CD3ζ, CD28, CD8, FcRIγ and less frequently, of CD4, CD7, OX40, and MHC(H2-Kb), the exact choice largely depending on the neighboring spacer and intracellular sequences [71]. It was demonstrated that the transmembrane modules based on CD3ζ and FcRIγ ensure efficient incorporation of CAR into endogenous TCR. This trans-signaling allows CARs lacking ITAMs or signaling sequences altogether to remain functional [69, 72-74]. Hence, CAR designs that mediate CAR inclusion or exclusion from TCR, as well as the recruitment of additional co-receptors, will likely result in the activation of quantitatively and qualitatively distinct signaling pathways, which requires further research.

**The signaling (intracellular) module**

The role of the signaling module of CARs is to transduce the activation signal to a T cell as soon as the extracellular domain has recognized the antigen. In normal T-cells, activation begins with the phosphorylation of ITAMs in the cytoplasmic portion of the CD3ζ subunit of the TCR complex [75]. Thus, in most CAR designs implemented to date, signaling sequences from CD3ζ are used as a module that triggers cell lytic activity. The ITAM-containing domains of other signaling subunits (e.g., FcRγ) were earlier tested for this role [4]; however, they proved to be less efficient in activating the cytotoxic function of CAR T cells [76, 77]. Induction of activating signaling in native T cells involves several steps. First, activated LCK kinase phosphorylates ITAM motifs in the cytoplasmic tail of CD3ζ, thereby activating ZAP-70 kinase, which simultaneously triggers several signaling cascades. These events are known as “signal 1.” Yet, to achieve complete T-cell activation, “signal 2” is also needed [78]. Signal 2 is typically provided by costimulatory receptors, such as CD28, whose binding to CD80/CD86 activates PI3K and triggers the PI3K-dependent signaling pathway. This, in turn, initiates the mTOR cascade and launches T cell proliferation.

Hence, in experiment, first-generation CARs, which contained the CD3ζ chain only, sent exclusively signal 1 to the cell. This led to a cytotoxic reaction against tumor cells [79] but did not provide enhanced proliferation of activated CAR T cells. In principle, signal 2 could potentially be provided by the native co-receptors present in the CAR T cells; however, many tumors do not express the corresponding ligands. In 1998, H.M. Finney and coauthors proposed the design of so-called second-generation CARs with a cytoplasmic domain additionally containing the costimulatory CD28 domain, fused together with CD3ζ, to overcome this difficulty. This CAR design provides both signal 1 and signal 2 to the T cell; as a result, the cell is activated, it destroys the target, and proliferates [58, 80, 81]. Besides CD28, signaling sequences from costimulatory receptors, such as CD134 (TNFRSF4, OX40), CD154 (CD40L), CD137 (4-1BB), ICOS (CD278), CD27, CD244 (2B4), etc., were successfully tested in CARs [82-88]. The nature of the costimulatory sequences (whether they are members of the IgSF or TNFRSF subfamilies) used directly influenced the phenotype and activity of CAR T cells [82, 89]. Further progress in the design of CAR signaling domains was based on combining two or more costimulatory sequences (4-1BB-CD28-CD3ζ being the most frequent one). These receptors, known as third-generation CARs, secrete a broader range of cytokines (including TNFα, GM-CSF, and IFNγ), are less susceptible to activation-induced cell death, and show higher efficacy in tumor elimination in mouse models [90-92]. Despite these promising pre-clinical findings, whether third-generation CARs are similarly more active in clinical conditions remains to be shown [93].

Second-generation CARs with the CD28-CD3ζ or 4-1BB-CD3ζ sequence still remain the most frequent CAR formats used in clinical practice [94-97]. Clinical and preclinical studies have demonstrated that CD28- CD3ζ-based CARs provide explosive expansion of CAR T cells *in vivo*, although this is also accompanied by CAR T cell exhaustion and terminal differentiation. In turn, this may lead to their limited persistence and a lack of antitumor effect [8, 98]. The dynamics of proliferation of 4-1BB-CD3ζ-containing CAR T cells is smoother: the 4-1BB-domain triggers a different activation pathway and alleviates the effect of a premature exhaustion of CAR T cells. Therefore, 4-1BB-CD3ζ CAR T cells persist in the organism for much longer, thereby providing a more durable and potent tumor control [87, 89, 99, 100]. Interestingly, Z. Zhao and coauthors have recently reported that providing 4-1BB-mediated co-stimulation in the context of CD28-CD3ζ- CARs (via co-expression of 4-1BB ligand) combines the advantages of both pathways and outperforms the conventional CAR designs, including third-generation 4-1BB-CD28-CD3ζ-containing CARs [101]. A similar approach based on the small-molecule controlled co-stimulatory switch to enhance the functionality of CAR T cells is used in the GoCAR-T-technology (Bellicum Pharmaceuticals). According to the data reported by the company, co-expression of the iMyD88-CD40 (iMC) hybrid molecule and the first-generation CAR engages a broader range of activation mechanisms, which results in more vigorous proliferation of GoCAR-T-cells that eliminate tumor cells both *in vitro *and *in vivo*.

It seems that there is no “one-size-fits-all” solution to CAR engineering, since different combinations of signaling and costimulatory modules are optimal for treating different types of cancers and these CAR variants are usually identified through trial and error. In this regard, the study by Australian researchers is notable: they have constructed a combinatorial library of the cytoplasmic domains of CAR using 14 signaling modules (CD3ζ, CD28, 4-1BB, CD27, DAP10, etc.) assembled in-frame. This library of CARs having an identical antigen-recognition moiety yet distinct signaling sequences was expressed in Jurkat T cells, and so CAR variants inducing the most potent cell activation were screened for. As a result, an unusual combination of the signaling sequences DAP10-CD3ζ-CD27 was identified, which was more effective *in vitro *than the CD28-CD3ζ [102]. L. Alvarez-Vallina and colleagues proposed a conceptually similar approach for identifying the optimal/novel antigen-recognition domains within CARs. They developed a lymphocyte display platform wherein scFv libraries are directly screened in the context of CAR T cells [103]. In this case, the scFv library in the CAR format is cloned into a lentiviral vector and expressed on the T-cell surface following viral transduction. The resulting library of scFv– CAR T cells is incubated with cells carrying the desired target, and T cells whose CARs are specific enough to recognize that target are collected and analyzed following several rounds of activation/selection and counter-selection. Selection of such CAR T cells is performed based on the activation markers that appear on the cell surface after CAR engagement (they become CD69- positive). In this approach, scFvs can be selected according to their ability to induce the activation and proliferation of scFv-CAR T cells rather than according to their affinity to the target. Hence, a key advantage of CAR T cell display is that CARs are selected right in the context of the synapse between the CAR T cell and the target cell, which may turn out to be more straightforward compared to the standard *in vitro *selection of high-affinity antigen-recognition binders, inevitably followed by their optimization and structural modification in the context of CAR T cells. Yet, one should bear in mind that CAR T cell display is associated with an important engineering constraint: namely, the significant decrease in the complexity of the CAR library amenable for screening (below 106–107). These studies show that assays that recapitulate the *in vivo *situation as close as possible should be used for testing CAR designs early on, since the CARs shown to perform well *in vitro *do not necessarily work in mice, nor do they by any means guarantee the same will be observed in a clinical setting. For this reason, it is currently believed that designing and testing the broadest range of CAR variants possible may be the only way to ultimately bring, at least, one of them to patients.

## CONCLUSIONS

The clear translational potential of the CAR T-cell platform has attracted interest to this field and prompted the development of various CAR designs. Nonetheless, the available experimental, and especially clinical, data that explore how the CAR structure affects its *in vivo* properties and which modifications ensure the maximum clinical effectiveness of CAR T-cell therapy remain scarce. Impressive results in the CAR T-cell therapy of rALL patients have stimulated attempts to adapt this platform to the treatment of solid cancers, and the first results indicate that further technological improvements are needed. Clearly, the widespread use of this platform will require additional systematic research and a more thorough understanding of the entire spectrum of the mechanisms that contribute to the establishment and maintenance of antitumor immunity.

## Abbreviations

CAR: chimeric antigen receptor
TCR: T-cell receptor
mAb: monoclonal antibody
scFv: single-chain variable fragment
scFv-CAR: scFv-based CAR
DARPin: designed ankyrin repeat protein
FITC: fluorescein isothiocyanate
VHH: camelid single-domain antibody (nanobody)
VLR: lamprey variable lymphocyte receptor
MHC: major histocompatibility complex proteins
ITAM: immunoreceptor tyrosine-based activation motif
